# Biological synthesis of hybrid silver nanoparticles by *Periploca aphylla* Dcne. From nanotechnology to biotechnology applications

**DOI:** 10.3389/fchem.2022.994895

**Published:** 2022-11-23

**Authors:** Saba Arshad, Natasha Anwar, Mamoona Rauf, Zeeshan Anwar, Mohib Shah, Muhammad Hamayun, Jalal Ud-Din, Humaira Gul, Sahar Nasim, In-Jung Lee, Muhammad Arif

**Affiliations:** ^1^ Department of Biotechnology, Abdul Wali Khan University Mardan, Mardan, Pakistan; ^2^ Department of Chemistry, Abdul Wali Khan University Mardan, Mardan, Pakistan; ^3^ Department of Botany, Abdul Wali Khan University Mardan, Mardan, Pakistan; ^4^ Department of Pharmacy, Abdul Wali Khan University Mardan, Mardan, Pakistan; ^5^ Department of Botany, University of Malakand, Totakan, Pakistan; ^6^ Department of Applied Biosciences, Kyungpook National University, Daegu, South Korea

**Keywords:** *Stevia rebaudiana*, Pe-AgNPs, PEGMA capped AgNPs, callus culture, bioactive compounds, antioxidants, secondary metabolites, green-synthesized silver nano particles

## Abstract

Nanotechnology is one of the advanced technologies that have almost universal implications in every field of science. The importance is due to the unique properties of nanoparticles; however, green synthesized nanoparticles are considered eco-friendly. The current project was rationalized to prepare green-synthesized biogenic *Periploca aphylla* Dcne. silver nanoparticles (Pe-AgNPs) and poly (ethylene glycol) methacrylate coated AgNPs nanocomposites (PEGMA-AgNPs) with higher potential for their application in plant tissue culture for enhancing the biomass of *Stevia rebaudiana* calli. The increased biomass accumulation (17.61 g/3 plates) was observed on a medium containing virgin Pe-AgNPs 40th days after incubation, while the maximum increase was found by supplementing virgin Pe-AgNPs and PEGMA capped AgNPs (19.56 g/3 plates), compared with control (12.01 g/3 plates). In this study, PEGMA capped AgNPs supplementation also induced the maximum increase in total phenolics content (2.46 mg GAE/g-FW), total flavonoids content (3.68 mg QE/g-FW), SOD activity (53.78 U/ml protein), GSH content (139.75 μg/g FW), antioxidant activity (54.3 mg AAE/g FW), FRAP (54 mg AAE/g FW), and DPPH (76.3%) in *S. rebaudiana* calli compared with the control. It was concluded that virgin Pe-AgNPs and PEGMA capped AgNPs (hybrid polymer) are potent growth regulator agents and elicitors that can be exploited in the biotechnology field for growth promotion and induction of essential bioactive compounds and secondary metabolites from various commercially important and medicinally valuable plants such as *S. rebaudiana* without laborious field cultivation.

## Introduction

Nanotechnology is quickly developing in practically all sectors of science and technology due to the unique chemical, physical, and biological features of nanoparticles (NPs). However, nanotechnology provides huge benefits to agriculture in terms of disease control and crop yield. Although nanoparticles have been studied for seed germination and other plant development characteristics, data on their effects on *in vitro* callus culture is limited. In such *in vitro* callus culture proliferation, NPs might be utilized as growth inducers to boost callus biomass and vigour by increasing the synthesis of important secondary metabolites and antioxidants, which are generally generated in limited amounts in parental plants (Anjum et al., 2019; Dehghani-Aghchekohal et al., 2022).

Silver nanoparticles (AgNPs) are being used in biological systems as part of the advancement of bionanotechnology (Hosny et al., 2022). This field provides approaches for solving challenges associated with food crop production by suppressing microbial agents (Mousavi and Rezaei, 2011; Pokhrel et al., 2012; Hussain et al., 2018). AgNPs have been employed in plants to stimulate germination, boost agricultural yields, and promote development (El-Batal et al., 2016; Kim et al., 2017). Some investigations have concentrated on exploring the role of AgNPs for antimicrobial activity and hormesis under *in vitro* conditions, in which development changes and increased biomass production have been observed (Parveen and Rao, 2015; Bello-Bello et al., 2017; Spinoso-Castillo et al., 2017; Saha and Gupta, 2018). The hormetic action is defined by low-dose growth promotion and high-dose inhibition. AgNP concentration, size, shape, chemical composition, reactivity, coating type, and aggregation levels, on the other hand, may influence plant development (Iavicoli et al., 2015; Calderón-Jiménez et al., 2017; Cvjetko et al., 2017; Pradas del Real et al., 2017). However, the processes of AgNP absorption, transport, accumulation, and mode of action in plants have received little attention.

AgNPs have gotten a lot of interest among the various metal nanoparticles (NPs) because of their appealing form, size, and environment-dependent characteristics that are completely different from those of bulk materials (Rajeshkumar and Bharath, 2017; Anwar et al., 2021). Although there are various chemical, physical, and biological approaches that have been utilized to synthesize AgNPs. Physical and chemical methods require energy-intensive, multi-step processes or toxic chemicals, so they have many limitations. These methods include chemical reduction, co-precipitation, seeding, microemulsion, inverse microemulsion, hydrothermal method, and sonoelectro-deposition. Nam and Luong (2019) suggest that co-precipitation is the preferable approach to producing nanoparticles. However, unfortunately, most of them use hazardous and toxic compounds, posing a substantial risk of toxicity. The characteristics of nanoparticles are also heavily influenced by their manufacturing process, temperature, and capping surfactants (Stadler et al., 2019).

Nowadays, green synthesized NPs are more environmentally friendly and cost-effective. Green chemistry, which uses plants as a source of AgNPs, is gaining popularity. Scientists are interested in using plant extracts to make NPs because it is a rapid, cost-effective, and ecologically friendly technique (Amooaghaie et al., 2015; Acharya et al., 2019).

Because all plants have medicinal properties, they are low in toxicity. The other reason is that traditional nanoparticle synthesis methods typically need dangerous reductants like sodium borohydride or hydrazine, as well as several stages in the synthesis process, including heat treatments, which sometimes result in hazardous by-products. Many researchers are also attempting to make ENMs and their production processes safer for people and the environment. They also look ahead to applications like the targeted delivery of chemotherapy drugs, tiny foodborne contaminant sensors, and advanced air-and water-filtration systems as plausible advances that could truly benefit society (Kessler 2011).

Recently, Erythrina suberosa (Mohanta et al., 2017), Phoenix dactylifera (Oves et al., 2018), Malva sylvestris (Esfanddarani et al., 2017), and Phyllanthus emblica (Esfanddarani et al., 2017) flowers have been effectively utilized to fabricate numerous types of AgNPs (Masum et al., 2019).

Previous reports showed that plant extracts can be utilized to produce AgNPs (Ahmad et al., 2021a). It was because plant phytochemicals, such as polyphenols, flavonoids, organic acids, alkaloids, and other antioxidant components, exhibited significant reduction and stabilization (Mohamad et al., 2014; Park, 2014). As a result, harnessing alfalfa extracts to synthesize AgNPs is entirely feasible. In terms of biological consequences, the various forms revealed unique physical and chemical features (Bondarenko et al., 2013). Furthermore, silver is not a vital nutrient for plants, and its toxicity to plants at high concentrations is well documented. As a result, we attempted to investigate how this synthesis may reduce toxicity while also demonstrating how metal NPs influenced plants.

The biocompatibility of nanoparticles with biological agents also influences their biological properties (Moulton et al., 2010). The three primary principles for producing nanoparticles using a green synthesis process are the solvent medium (ideally water), an ecologically friendly reducing agent, and a nontoxic chemical for nanoparticle stabilization (Song et al., 2022).

*S. rebaudiana* is agriculturally and pharmaceutically significant plant all over the world. It is a member of the sunflower family (Asteraceae). Because of its sweet flavor, it is also known as a sweet leaf or sugar leaf (Ahmad et al., 2021b). *S. rebaudiana* is well-known for its sweet flavor due to the chemical components found in the leaves of Stevia, such as Steviol glycosides, which frequently contain stevioside and rebaudioside (Ahmad et al., 2020). *S. rebaudiana* is used to treat hypotension and hypoglycemia, and its active constituents (Steviol glycosides) are naturally non-carcinogenic and one of the finest sucrose alternatives (Geuns, 2003; Jeppesen et al., 2003). It is also employed as a natural scavenger or antioxidant to eliminate free radicals from the body. Its leaves or active chemicals can be utilized to treat cardiac disease and are also beneficial in the treatment of obesity and diabetes (Hwang, 2006). In addition to its sweetening characteristics, *S. rebaudiana* contains natural features that make it anti-pathogenic (Fazal et al., 2011), anti-cancerous, tooth decay inhibitor (Dey et al., 2013), anti-hypersensitive, and free radical scavenger properties (Ahmad et al., 2011a). There are several techniques for cultivating *S. rebaudiana*, including seed germination and stem cuttings. The stem-cutting method propagates fewer plants because of the high need for primary stock, which is time-consuming and season-dependent. Because of their tiny size, seed germination yields low biomass of plantlets, and seeds often lose their capacity to germinate (immature embryos) after collecting (Arpita et al., 2011). Various approaches are employed to obtain the needed and useful bioactive chemicals and biomass in a short period. One biotechnological technique employs plant cells, tissue, and organ cultivation to protect endangered species while producing more biomass in a shorter period (Abbasi et al., 2012; Aman et al., 2013). Compared with conventional propagation, the callus culture of *S. rebaudiana* and other medicinal plants produces consistent amounts of secondary metabolites and is also easier to develop. Callogenesis is the best suitable alternative for optimum production of biomass and secondary metabolites since it is a valuable approach compared to several *in vitro* methods. Therefore, the rationale for this study was to attain the efficient callogenesis of *S. rebaudiana* with feasible scaled-up for maximum biomass, antioxidants, and bioactive compound induction. Recently, the influence of nanotechnology in the field of agricultural sciences has been explored and implied with a more in-depth investigation to propagate and cultivate plant species of agricultural relevance.

The primary goal of this study was to produce biogenic, green-synthesized AgNPs in a single step using a *Periploca aphylla* Dcne. extract (a member of the Asclepiadaceae family that grows in arid climates). *Periploca aphylla* Dcne. is administered by indigenous medicine practitioners and the local populace as a stomachic, tonic, anticancer, antiulcer, and for the treatment of inflammatory illnesses. Plants or their extracts have long been used in traditional health care systems to treat a variety of illnesses, including diabetes.

**FIGURE 1:**
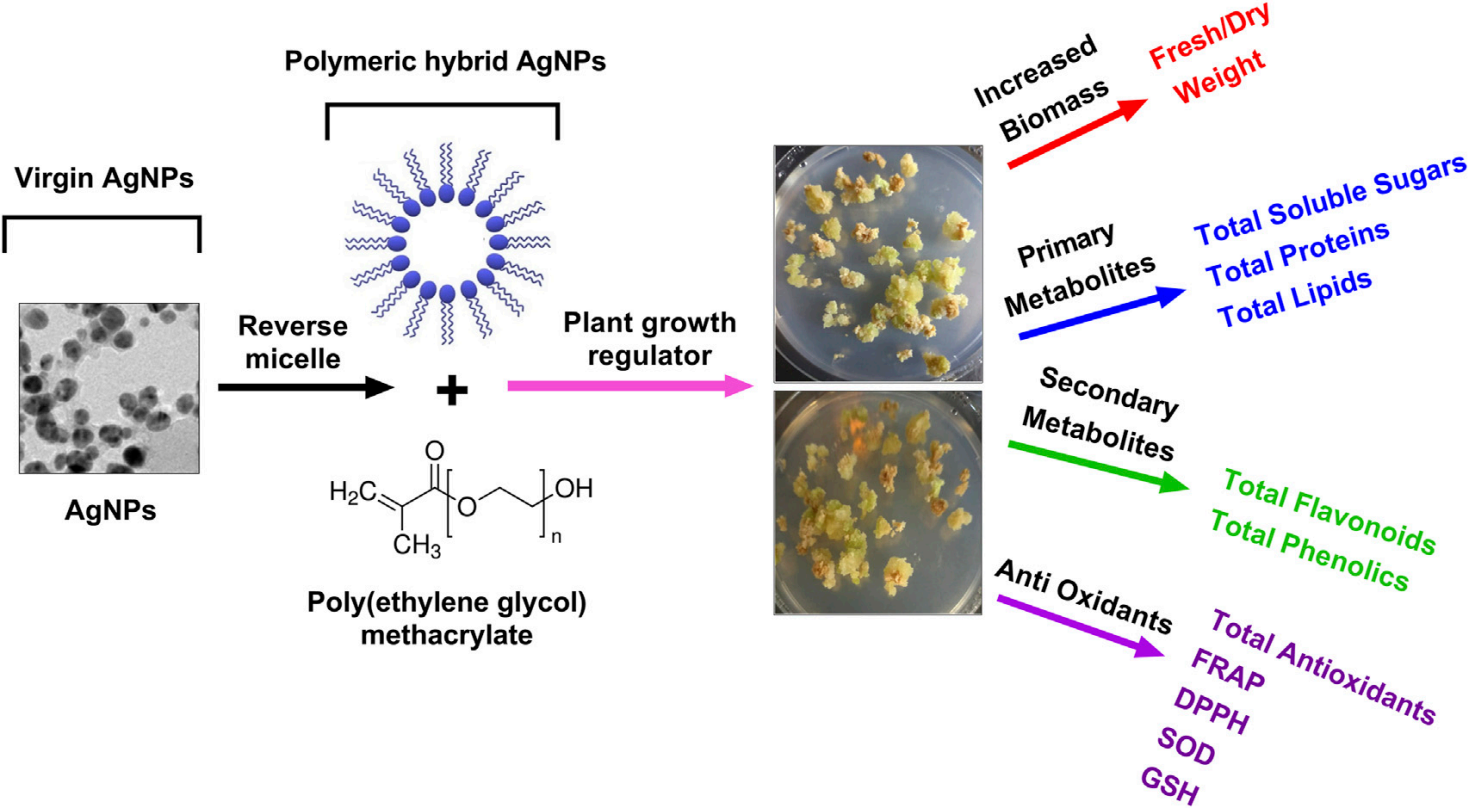
A schematic plan of the experimental setup of PEGMA capped AgNPs and their applications in plant callus culture, *in vitro.*

The current research was rationalized and aimed to synthesize the biogenic AgNPs and characterize the physicochemical properties of Pe-AgNPs and poly (ethylene glycol) methacrylate coated AgNPs nanocomposites (PEGMA-AgNPs) using different techniques, such as XRD, FTIR spectroscopy, SEM, TEM, EDS, ZP, and DSL analysis were also investigated to explore their biomedical potential. Moreover, the current research was focused on exploring the effect of green synthesized AgNPs formulations (virgin Pe-AgNPs and polymer hybrid PEGMA capped AgNPs) on the stevia (*S. rebaudiana*) callogenesis response to produce biomass, antioxidants, and bioactive compounds, *in vitro* (Figure 1).

## Materials and methods

### Plant material, chemicals, equipment, and reagents

*Periploca aphylla* Dcne., a well-known traditional medicinal plant in Pakistan’s northern territory, was collected in the district of Mardan (located between 34° 05′ and 34° 32′ north latitudes and 71″ 48′ to 72° 25′ east longitudes). All the reagents purchased and used were of analytical grade. AgNO_3_, chloroform, ethanol, benzene, n-hexane, distilled water, and Triton X-100 were purchased from Merck (Darmstadt, Germany). All UV-visible spectra were obtained using a Shimadzu UV-2550 spectrophotometer and a quartz cell with a path length of 1 cm. Fourier transform infrared (FTIR) spectra were obtained using KBr plates and Nicolet 6700 FT-IR equipment (Thermo Scientific) by placing a drop of sample dispersion and drying the sample. The nanoparticles and polymer hybrid solution were poured onto copper grids to prepare specimens for transmission electron microscopy (TEM) examination in a JEM 2100F coupled with a 200 kV field-emission gun. The sample solution was filtered with a Millipore syringe filter (0.2 m pore size). The dynamic light scattering (DLS) analysis was done using a Brookhaven Equipment Corporation model BI-200SM instrument. The X-ray diffraction was performed using a JEOL JDX 3532 X-ray diffractometer (Lab tech., Yokosuka, Japan).

### Preparation of *Periploca aphylla* Dcne. plant extract

After boiling 15 g of dry tissue in 400 ml of distilled water for 15 min, the extract was filtered. The filtered plant extract was kept at room temperature for future usage, with a shelf life of 2 weeks.

### Preparation of AgNO_3_ solution and poly (ethylene glycol) methacrylate-coated silver nanoparticles

In a 100 ml volumetric flask, 1 mM AgNO_3_ solution (Merck, Darmstadt, Germany) was made using distilled water. Different salt-to-plant extract ratios were used to combine the prepared silver solution and plant extracts (1:1, 2:1, 3:1, 4:1, 5:1, 6:1, and 7:1 ml). The surface modification was started by mixing 5 mg of NPs with 1 ml of Triton X-100, 10 ml of n-hexane, and 5 ml of benzene (Merck) mixture (7:3), and then sonicating for 5 min. After that, the solution was mixed for 1 h before adding a poly (ethylene glycol) methacrylate (PEGMA, Mn526) (Sigma-Aldrich, Milwaukee, WI) solution (540 μl of PEGMA dissolved in 100 μl water) and stirring it for 24 h. The solution was then diluted with ethanol until it reached 45 ml and centrifuged for 30 min at 14,000 rpm. The pellets were retrieved by removing the supernatant and solvent. This process was repeated thrice to eliminate the unreacted silver salt and unspecific metabolites and compounds of plant extract. The resultant AgNPs were used for characterization, as well as accessing callus culture development and biochemical profiling in *S. rebaudiana*.

### Characterization of virgin *P. aphylla* Dcne. silver nanoparticles and poly (ethylene glycol) methacrylate-capped silver nanoparticles

The generated virgin Pe-AgNPs and PEGMA-capped AgNPs were morphologically and morphometrically evaluated by adapting the method as described by Asad et al. (2022), using a quartz cell with a 1 cm path length and a UV-visible single beam Shimadzu UV-2550 spectrophotometer (Shimadzu Corporation, Kyoto, Japan). The X-ray diffraction pattern of Ag nanoparticles were analyzed using a JEOL JDX 3532 X-ray diffractometer (Lab tech., Yokosuka, Japan). Fourier transform infrared (FTIR) spectra were obtained using KBr plates and Nicolet 6700 FT-IR equipment (Thermo Fisher Scientific, Waltham, MA) by placing a drop of sample dispersion and drying the sample. The virgin Pe-AgNPs and PEGMA capped AgNPs solutions were dropped onto copper grids to prepare specimens for transmission electron microscopy (TEM) observation in a JEM 2100F (Jeol Ltd., Tokyo, Japan) equipped with a 200 kV field-emission gun. Dynamic light scattering (DLS) analysis was carried out using a Brookhaven Equipment Corporation model BI-200SM instrument.

### *In vitro* callus growth assay of *S. rebaudiana* and experimental design

Healthy stevia plants were recruited from the Ternab Agriculture Research Centre, Peshawar, Pakistan. The branches were cut into axillary buds measuring around 1.5 cm in length. MS growth media was used for callus cultures along with supplementation of NAA, BAP, 2,4-D, virgin Pe-AgNPs, and PEGMA-capped AgNPs. The cultured cultures were kept at 25°C with 16 h of light and 8 h of darkness and relative humidity of 72–75%.

The stock solution of virgin Pe-AgNPs and PEGMA-capped AgNPs was made according to the Asad et al. (2022) methodology. All calli cultures were grown in the growth chamber (light intensity of 40–50 mol m^2^ s^1^ at 25°C, photoperiod 16/8 h) until phenotypic variations in biomass among treatments were visible. The information was gathered to assess the impact of virgin Pe-AgNPs and PEGMA-capped AgNPs on fresh weight (FW), dry weight (DW), primary metabolites, secondary metabolites, and antioxidant capacity. Poly (ethylene glycol) methacrylate was used as a negative control. The experimental design was completely randomized, where *in vitro* cultures were established and segregated randomly into three groups of at least 10 replicates each.

The following four treatments were designed for the experimental setup.

Treatment 1: Control (2 mg/L NAA + 2 mg/L BAP + 2 mg/L 2,4-D in MS media).
Treatment 2: Pe-AgNPs (2 mg/L Pe-AgNPs + 2 mg/L NAA + 2 mg/L BAP + 2 mg/L 2,4-D in MS media).
Treatment 3: Polymer hybrid (2 mg/L PEGMA-AgNPs + 2 mg/L NAA + 2 mg/L BAP + 2 mg/L 2,4-D in MS media).

### Evaluation of biomass production from *in vitro* callus cultures

Callus biomass was recorded 40 days after the first inoculation. To determine FW, the aggregate of calli from all treatments was gathered, rinsed with sterilized distilled water, and dried between the filter papers to eliminate external moisture. Each calli aggregate was oven dried (60°C) for 20 h for DW.

### Metabolic analysis for assessment of primary and secondary metabolites

**Total soluble sugars:** TSS were determined according to the method of Nayer and Reza (2008) as explained by Aziz et al. (2021a) by homogenizing 0.5 g of fresh tissue (in 10 ml of distilled water). Absorbance was taken at 520 nm.

**Total lipid content:** TLC was determined as mentioned by Van Handel (1985) as explained by Ali et al. (2021).

**Total Protein Content:** TPC in callus cultures was determined according to Lowry et al. (1951) as explained by Rauf et al. (2022).

**Total phenolic content (TPC)**: Total phenolic compounds were assessed using the method given by Malik and Singh (1971), as stated by Aziz et al. (2021b). In a 100 ml conical flask, a known quantity of stevia leaf or callus powder was applied. To this, 25 ml of 0.3N HCl in methanol was added and shaken for an hour on an environmental shaker (Brunswick, United States) at 150 rpm.

The crude extract was filtered using Whatman No. 1 filter paper after shaking. In a hot water bath, the filtrate was evaporated to dryness. Hot water was added to the residue, and the final volume was adjusted to 100 ml with distilled water. A 1 ml sample was collected and placed in a test tube. This was then mixed with 1 ml of diluted Folin-Ciocalteu reagent and 35% sodium carbonate. After 10 min, 2 ml of distilled water was added, and the color intensity was measured at 620 nm in a UV spectrophotometer (Hitachi 220S) against a reagent blank. The total phenolic content was measured using a gallic acid-prepared standard curve.

**Total flavonoid content (TFC):** Total flavonoids were quantified in the manner described by Ali et al. (2020). In a 100 ml conical flask, a known amount of stevia leaf or callus powder was placed. To this, 25 ml of 0.3N HCl in methanol was added and shaken for an hour on an environmental shaker (Brunswick, United States) at 150 rpm. The crude extract was filtered using Whatman No.1 filter paper after shaking. In a water bath, the filtrate was evaporated to dryness. Hot water was added to the residue, and the final volume was adjusted to 100 ml with distilled water. A 1 ml aliquot was placed in a test tube, and 1 ml of 20% HCl and 0.5 ml of formaldehyde were added before the tubes were allowed to stand overnight.

### Biochemical analysis for assessment of antioxidant activities

**Sample preparation:** To make the aqueous extract collected in 100 ml conical flasks, 150 mg of freshly powdered stevia callus tissue was utilized. This was mixed with 50 ml of water and shaken for an hour on an environmental shaker (Brunswick, United States) at 150 rpm. After removing the flask, the contents were filtered *via* filter paper (Whatman No. 1). Without storing the filtrate, it was employed directly for FRAP, DPPH testing, and total antioxidant evaluation.

**FRAP assay:** The method proposed by Benzie and Strain (1996) was followed. The idea of this approach is based on the reduction of a ferric-tripyridyl-triazine complex to its ferrous, colored state in the presence of antioxidants. The freshly prepared FRAP reagent contained 2.5 ml of a 10 mmol/L TPTZ (2,4,6-tripyridyl-s-triazine, Sigma) solution in 40 mmol/L HCl, 2.5 ml of 20 mmol/L FeCl3, and 25 ml of 0.3 mol/L acetate buffer, pH 3.6. After incubating at 37°C for 10 min, aliquots of 40 ml of sample filtrate were combined with 0.2 ml of distilled water and 1.8 ml of FRAP reagent, and the absorbance of the reaction mixture at 593 nm was determined spectrophotometrically. Ascorbic acid was employed as a standard.

**DPPH assay:** The method for measuring the DPPH free radical scavenging effect was adapted from Germano et al. (2002). Methanolic and water extracts of Stevia callus were tested for their potential to donate hydrogen or scavenge radicals by utilizing the DPPH radical. For the experiment, 200 ml of filtrate was placed in test tubes, and the volume was increased to 1 ml with methanol. Three milliliters of newly produced DPPH (200 mM) solution in methanol were added to the sample tube and forcefully stirred for 15 s. The sample tube was then placed in a 37°C water bath for 20 min. The sample’s absorbance was measured using a UV spectrophotometer at 517 nm. As standard, gallic acid was employed. The free radical scavenging activity was calculated as a percentage of DPPH° discoloration by using the following equation.

% scavenging DPPH° free radical=100×(1−AE/AD).

In this equation, AE is the absorbance of the solution when a particular concentration of extract is added, and AD is the absorbance of the DPPH solution when nothing was added to this solution.

**Total antioxidant capacity:** TAC was calculated using fresh callus samples (3.0 g) crushed in methanol, as stated by Khan et al. (2022). The sample extract was treated with 3 ml of a solution containing H_2_SO_4_ (0.6 mM), sodium phosphate buffer (28 mM), and ammonium molybdate (4 mM). After incubation, absorbance at 695 nm was measured against a blank.

### Statistical analysis

The experiments were conducted using a completely randomized design. One-way analysis of variance (ANOVA) was done using SPSS ver. 16.0 software (Chicago, IL) (*p* ≤ 0.05).

## Results and discussion

### Optimization and characterization of *P. aphylla* Dcne. stabilized silver nanoparticles

Silver nanoparticle biosynthesis has been reported previously from bacteria such as Pseudomonas indica (Salem et al., 2022), endophytic fungus, Cryptosporiopsis ericae PS4 (Devi and Joshi, 2014), and king oyster, Pleurotus eryngii (Acay and Baran, 2019). Researchers have developed green biosynthesis of metallic nanoparticles, such as silver nanoparticles, that have been synthesized from various plants, including Salmalia malabarica, Opuntia dilleniid (Ahmed et al., 2022), Moringa oleifera (Moodley et al., 2018), Svensonia hyderabadensis (Rao and Savithramma, 2011), Allium cepa (Balamanikandan et al., 2015), Euphorbia hirta (Elumalai et al., 2010), and Buchu (Chiguvare et al., 2016). However, in the current study, we generated polymer/NPs composites by the *ex-situ* reverse micellization process by dispersing AgNPs directly into a polymer matrix to form composites with greater stability and solubility with no shape change or particle aggregation, utilizing a robust, economical, affordable, and an environmentally safe biological way for the synthesis of AgNPs (Pe-AgNPs) using *P. aphylla* Dcne. extract. The entire approach may be called “green-synthesized,” with huge benefits over chemically synthesized techniques, such as environmental friendliness, lack of harmful by-products, low temperature, and pressure.

**FIGURE 2:**
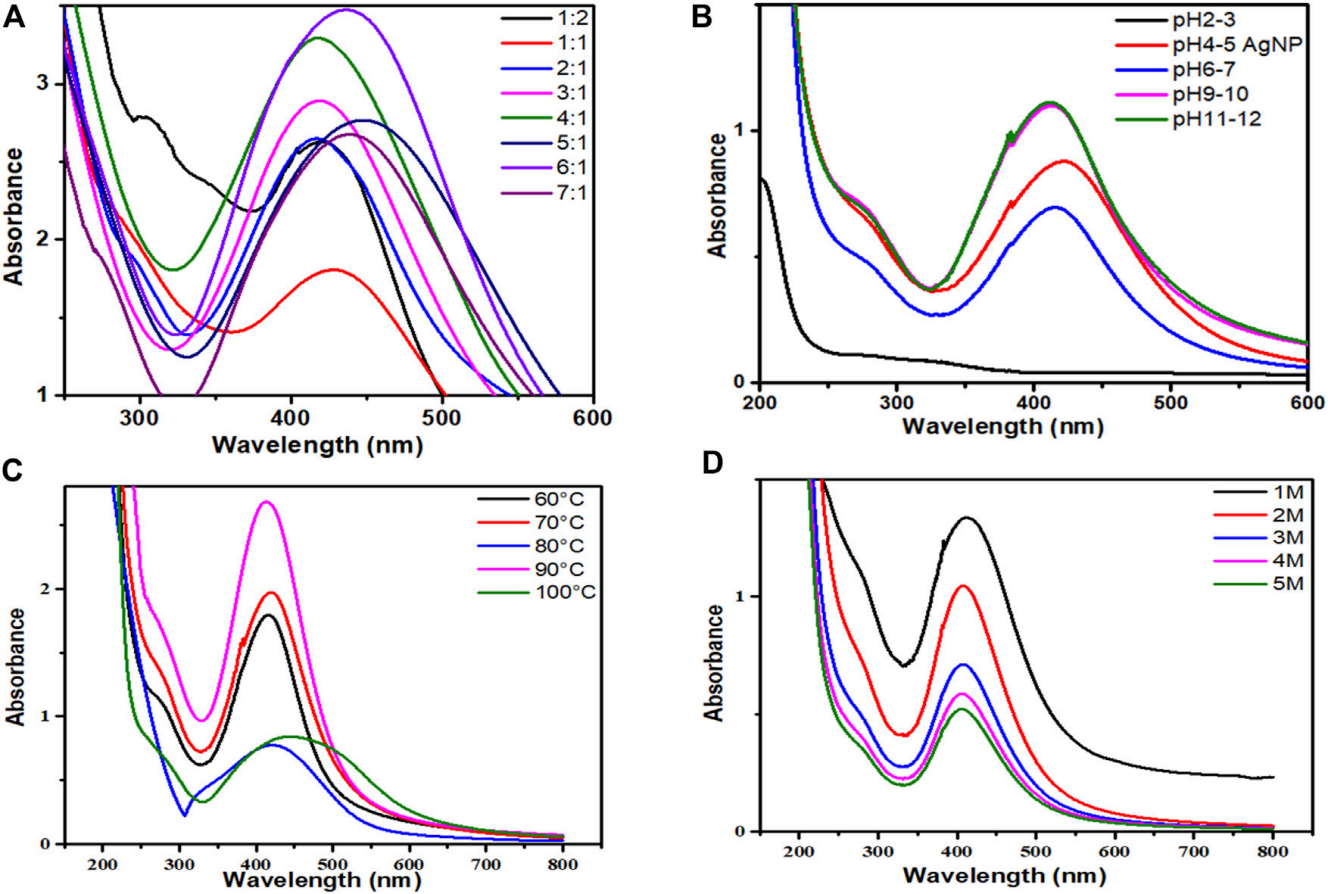
**(A)** UV-visible spectra of AgNPs/*Periploca aphylla* Dcne. having different AgNO_3_/Plant ratios, **(B)** Effect of pH on the stability of AgNPs, **(C)** Effect of temperature on the stability of AgNPs, **(D)** Effect of salt (NaCl) on the stability of AgNPs.

The ultraviolet-visible spectra of AgNPs capped with *P. aphylla* Dcne. revealed the highest absorbance peak at 425 nm. SPR peaks in the 400–500 nm range indicate the presence of AgNPs (Ahmad et al., 2011b). The salt-to-plant ratio was changed to maximize the reaction. The best-optimized ratio selected for future research was 4:1, which demonstrated high SPR in the typical area of AgNPs (Figure 2A).

The pH of the solution affects the stability of manufactured AgNPs. The pH variation affects the form and size of the particles. Figure 2B depicted the change in intensity and peak absorption wavelength when the pH of the solution was changed. When the pH was raised from 4 to 11, the absorption maxima shifted from 415 to 440 nm. The strength of absorption is also boosted. This demonstrates that preparing AgNPs with *P. aphylla* Dcne. the extract works best at pH 11. Temperature is thought to be an essential element in the creation of nano-sized particles.

While analyzing the impact of temperature, it was discovered that as the temperature increased from 60 to 90°C, the absorbance increased (Figure 2C). At around 90°C, maximum absorbance and a sharp peak were observed, indicating monodispersed small particles and a decrease in NP size. As the temperature rises above 100°C, the absorbance falls. Asymmetry with large peak width revealed that nanoparticle aggregation begins owing to the denaturation of protein molecules at high temperatures (Amin et al., 2012). As a result, 90°C was found to be the optimal temperature for the synthesis of nanoparticles in this scenario.

The stability of AgNPs is capped with *P. aphylla* Dcne. diminishes as the concentration of NaCl increases (Figure 2D). As the concentration of sodium chloride increases, the stability of silver NPs diminishes. Increased salt concentration causes AgNP aggregation owing to Cl^−1^ ions due to electrostatic attraction or repulsive interactions of silver ions with sodium chloride ions in water at constant pH. Changes in salt ionic strength may also influence the microenvironment of ions. As a result, the stability of AgNPs can alter the ions’ microenvironment and the stability of AgNPs (Naz et al., 2013).

### UV-Vis spectra and fourier transform infrared analysis for Pe-AgNPs and polymeric hybrid poly (ethylene glycol) methacrylate capped AgNPs

**FIGURE 3:**
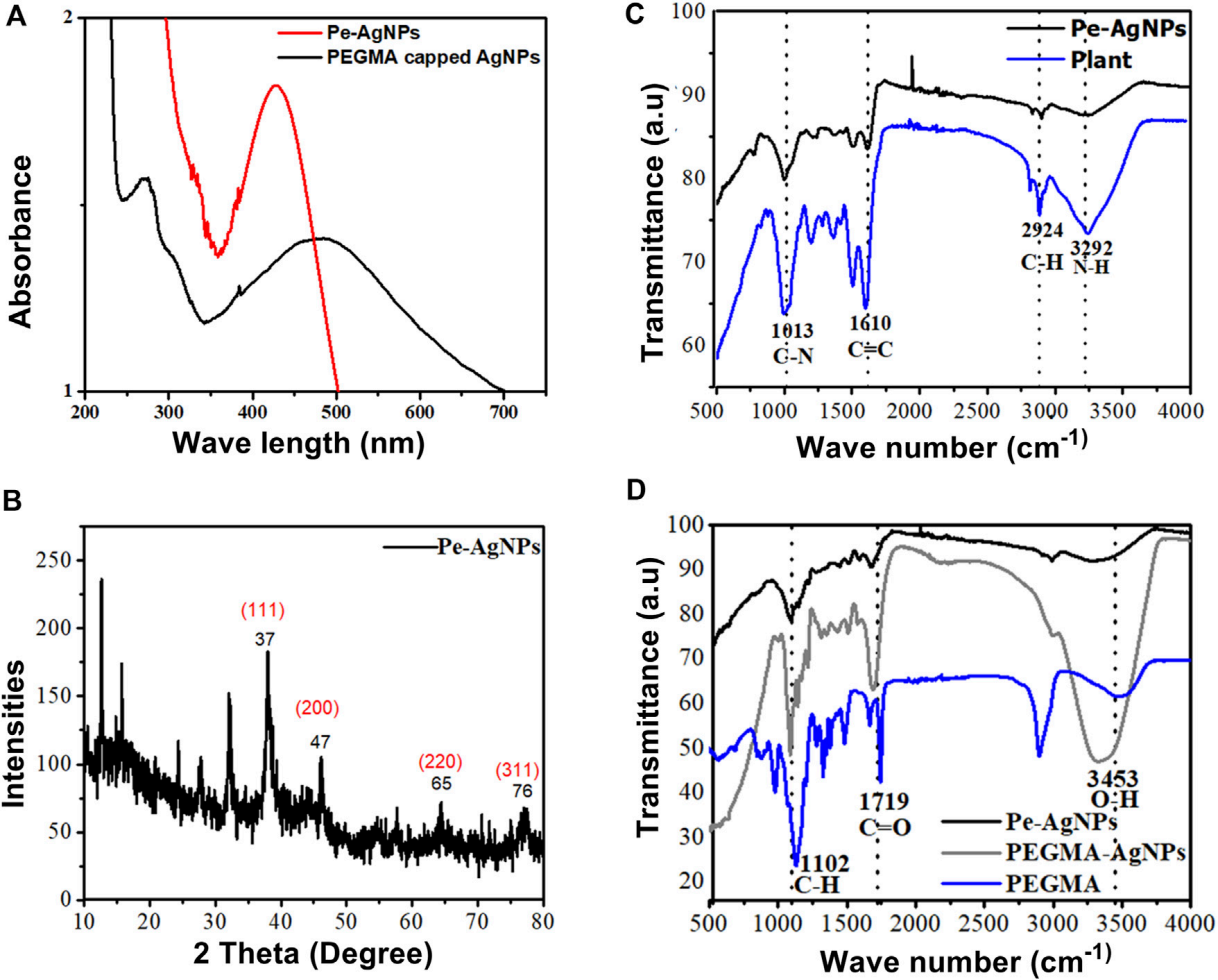
**(A)** UV-visible spectra of Pe-AgNPs and PEGMA capped AgNPs, **(B)** XRD of Pe-AgNPs, **(C)** FTIR Spectra of *P. aphylla* Dcne. plant extract and Ag-capped *P. aphylla* Dcne., **(D)** FTIR spectra of the PEGMA, Pe-AgNPs, and PEGMA capped AgNPs.

PEGMA, which is known to improve nanoparticle stability and biocompatibility, was used directly after production in coating nanoparticles. The SPR absorbance peaks were monitored using UV-Vis spectroscopy to investigate the effect of PEGMA coating on the stability of AgNPs solution (Figure 3A). Changes in the local nanoparticle environment affect both the position and amplitude of the peak. Because of the decrease in the interparticle distance, particle aggregation generates a red shift in the UV-vis absorption spectrum (Cho et al., 2008; Jones et al., 2011).

The SPR peak wavelength increased with polymer coating because the dielectric material around the particle changed, but the particles also grew slightly larger due to polymer adsorption on the surface. Thus, the peak position may be used to check the stability. The aqueous solution of *P. aphylla* Dcne. was phytochemically examined to determine its active components. *P. aphylla* Dcne. phytochemical examination revealed the presence of steroids, terpenoids, reducing sugars, tannins, beta cyanin, and amino acids (Rauf et al., 2013). X-ray diffraction crystallography was used to identify the molecular and atomic structures of crystals, in which crystal-shaped atoms caused a beam of accident x-rays to diffract in many specific directions. The crystallinity of dried AgNPs is capped by the extracts of *P. aphylla* Dcne. was confirmed with the help of X-ray diffraction. The X-ray diffraction pattern of silver nanoparticles showed Braggs representative of the face-center cubic structure of silver. The characteristic diffraction peaks of the fcc silver lattice for nanoparticles were at 37, 47, 65, and 77 in two thetas with corresponding planes at (111), (200), (220), and (311) respectively. The crystalline nature of *P. aphylla* Dcne. capped silver nanoparticles were confirmed by XRD analysis. The formation of lattice planes of nanoparticles was indicated by the XRD pattern, and the presence of small-sized nanomaterial and no evidence of the presence of bulk residue (remnant) and impurities were expressed by the peak broadening of this pattern (Keshari et al., 2020) (Figure 3B).

Adsorption peaks were seen in the FTIR spectra of PEGMA at 3,453 cm^−1^ (O-H), 2,869 and 1,102 cm^−1^ (C-H), 1719 cm^−1^ (C=O), and 1,628 cm^−1^ (C=C) (Figure 3C).

The FTIR spectra of *P. aphylla* Dcne. were used in the current investigation. Peaks were observed at 1,610, 2,924, and 3,292 cm^1^ (Figure 3D). After interaction with AgNO3, the peaks shift to a higher wave number and their intensity decreases, such as 1,630, 2,987, and 3,300 cm^1^ (Figure 3D).

Because of the C=O, the extract’s stretch at 1,610 cm^1^ is moved toward a higher wave number side at 1,630 cm^1^ (Anandalakshmi et al., 2016; Ismail et al., 2018). The smaller peak at 2,924 cm^1^ has been relocated to 2,987 cm^1^ due to the -CH stretch of alkanes. The band at 3,292 cm^1^ is associated with NH (amide) stretching (Narayanan and Sakthivel, 2008). Carbonyl groups confirm the existence of flavanones or terpenoids (Zia et al., 2016). Adsorption peaks were seen in the FTIR spectra of PEGMA at 3,453 cm^−1^ (O-H), 2,869 and 1,102 cm^−1^ (C-H), 1719 cm^−1^ (C=O), and 1,628 cm^−1^ (C=C) (Figure 3D).

The FTIR spectra of AgNPs were dramatically affected by PEGMA coating, particularly the C=O and C-H peaks from the methacrylate tail and the O-H peak from the opposite end. The FTIR spectra revealed that no new peak was discovered following the hybridization of Pe-AgNPs with PEGMA. However, after coating, there is a change in the location, intensity, and shape of the peak, indicating that the coating process happened by physicochemical adsorption rather than a real chemical reaction (Permadi et al., 2012).

### Scanning electron microscopy, transmission electron microscopy

**FIGURE 4:**
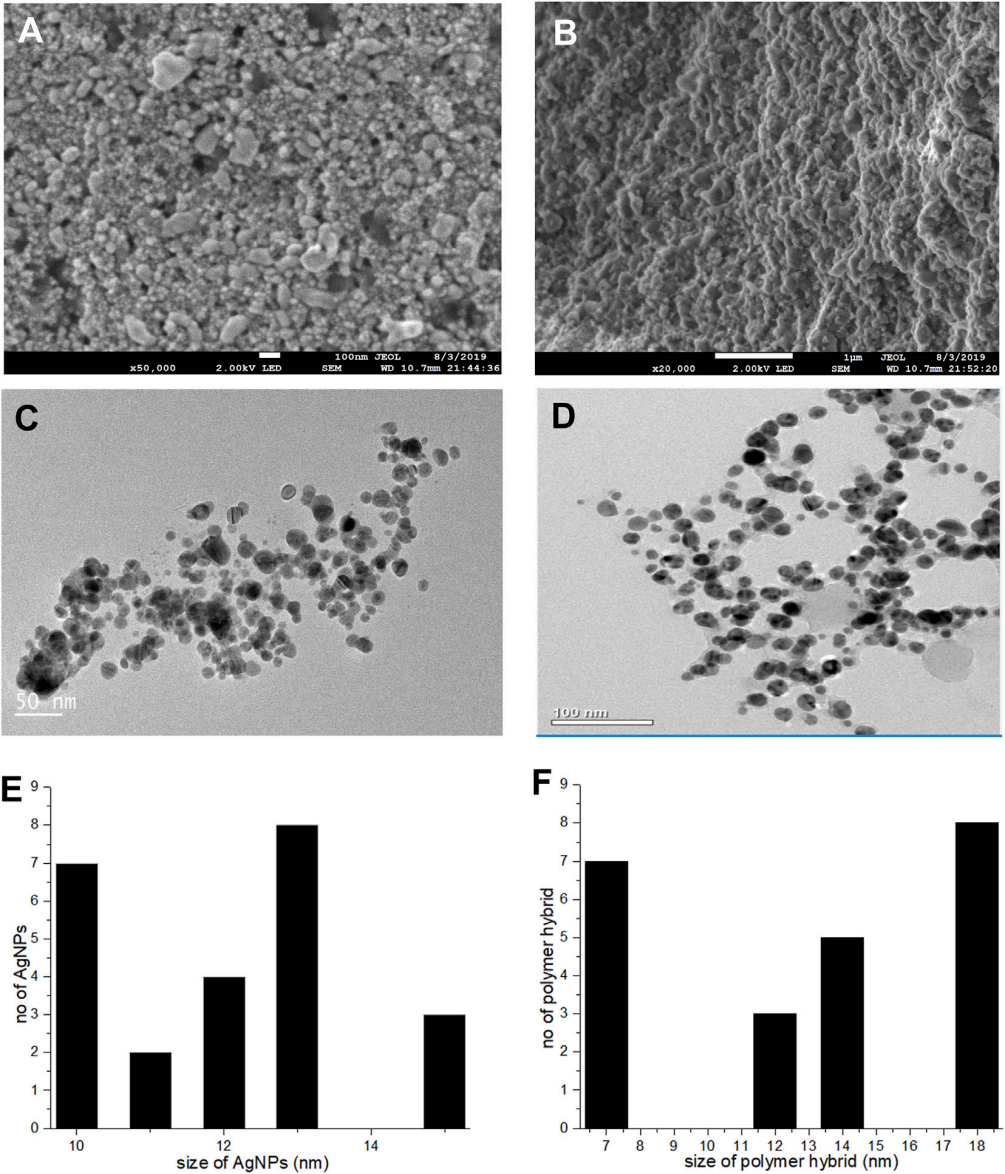
**(A)** SEM images Pe-AgNPs **(B)** SEM images PEGMA capped AgNPs, **(C)** TEM images Pe-AgNPs, **(D)** TEM images PEGMA capped AgNPs, **(E)** Histogram Pe-AgNPs and **(F)** Histogram PEGMA capped AgNPs.

A topographical perspective reveals that nanoparticles are spherical and crowded together. SEM scans of Pe-AgNPs revealed an average size of 10–60 nm (Figure 4A) (Jeevanantham et al., 2018). The SEM examination of PEGMA capped AgNPs, as shown in Figure 4B, clearly shows uniform dispersion of the NPs in a mesh-like structure created by the poly (ethylene glycol) methacrylate chains (Agbo et al., 2017). The nanocomposites range in size from 10 to 200 nm.

The porous character of the nanocomposites is depicted by the SEM. Because spherical NPs are particularly potent during biological activities due to their capacity to quickly penetrate, the observed nature of the SEM suggests that the NPs-polymer composites are very attractive for applications in bio-delivery and catalysis.

A TEM examination was also done to further analyze the sizes and dispersion of these NPs and NPs-polymer composites. The TEM scans revealed that the produced Pe-AgNPs are generally spherical (Figure 4C). Figure 4E depicts the average size of AgNPs, discovered to be 11.8 nm. The presence of PEGMA capping on the AgNPs is also confirmed by the TEM pictures. Figures 4D,F indicate that the polymer-capped silver nanoparticles were spherical with an average diameter of around 14.3 nm.

### Dynamic light scattering particle size distribution analysis

As predicted, the DLS measured size is somewhat bigger than the TEM recorded size because TEM measures the precise size and does not include any capping agents, but DLS measures the diameter of the particle plus any ions or molecules attached to the surface and moves with the AgNPs in solution (Huang et al., 2007; Cumberland and Lead, 2009; Samberg et al., 2009; Singhal et al., 2011).

**FIGURE 5:**
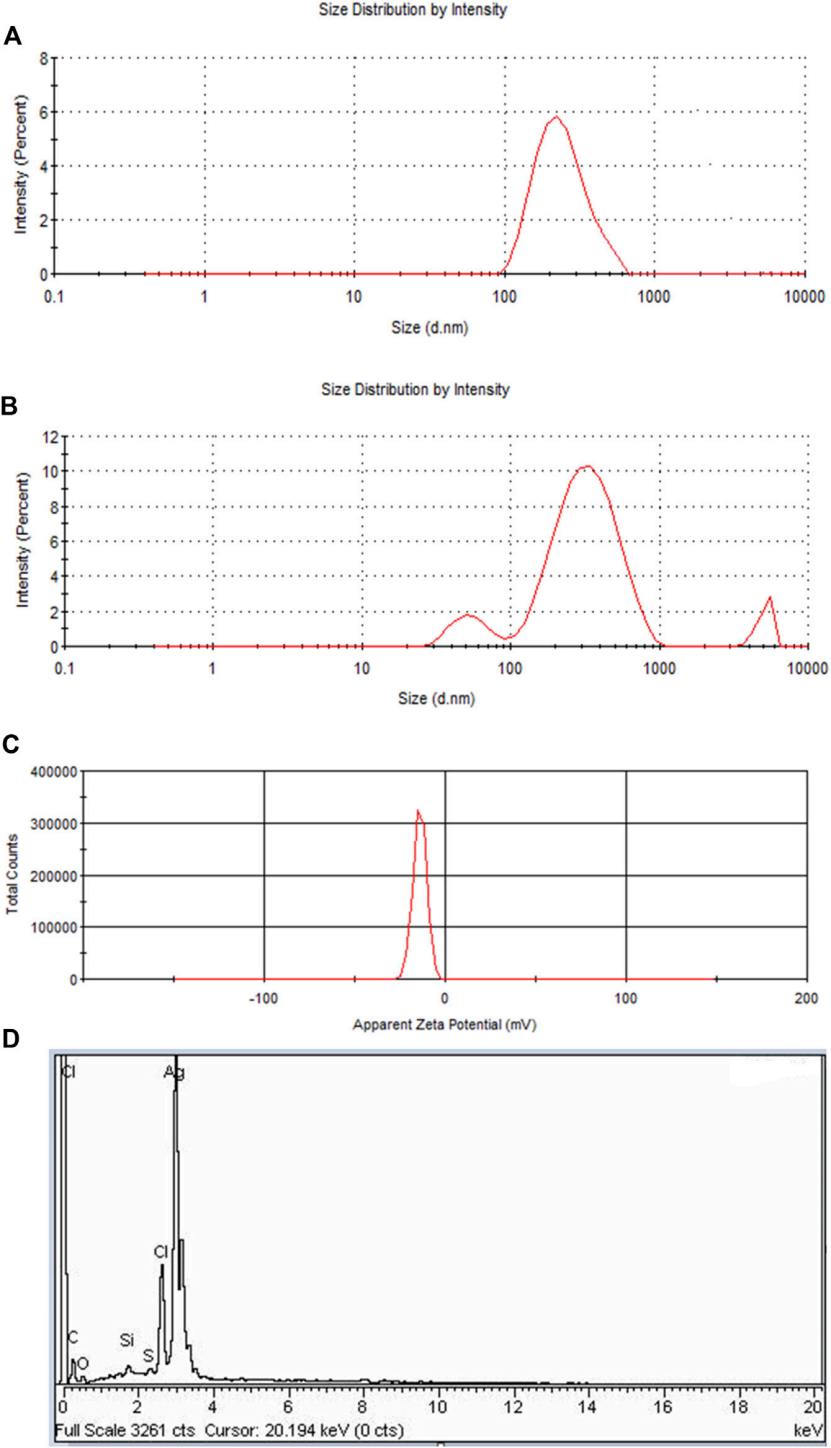
**(A)** DLS of Pe-AgNPs, **(B)** DLS of PEGMA capped AgNPs showing an increase in the size of NPs due to polymer capping, **(C)** Surface zeta potential of the PEGMA capped AgNPs, **(D)** EDS spectrum of PEGMA capped Pe-AgNPs.

Two peaks were produced for PEGMA-capped Pe-AgNPs at roughly 50 and 295 nm (Figures 5A,B). It demonstrates the presence of particles of various sizes in the colloid. The aggregation of the PEGMA-capped Pe-AgNPs is responsible for the rise in amplitude at 295 nm and the concomitant drop in peak intensity at 50 nm. The agglomerated component has a diameter of approximately 347 nm, whereas the unagglomerated counterpart has a diameter of around 50 nm (Banerjee, et al., 2015).

### Zeta potential and energy dispersion spectroscopy

If all the particles in suspension have a large negative or positive Zeta potential (ZP), then they will tend to repel each other and there will be no tendency for the particles to come together (Dave et al., 2017). A negative Z.P-value of about −20.2 mV was observed for PEGMA-capped AgNPs. The higher surface charge of these prepared PEGMA-capped AgNPs, as indicated by the Z.P-value (Figure 5C), correlates well with their higher degree of stability (Honary and Zahir, 2013).

The EDX signal indicates the presence of Ag, C, and O as shown in Figure 5D. In EDX analysis of PEGMA-capped Pe-AgNPs, the percent weight of silver is 74.77% and that of carbon is 9.5%. The existence of C and O indicated organic compounds were incorporated into the nanoparticles, which is ascribed to poly (ethylene glycol) methacrylate (Ding et al., 2019).

### *In vitro* growth response of *S. rebaudiana* upon silver nanoparticles supplementation

The addition of NPs can result in increased bioactive component content in the cell, shoot, and root cultures, perhaps due to the generation of reactive oxygen species (ROS), activation of antioxidant enzymes, and regulation of particular genes. However, the mechanism underlying this is yet unknown. A variety of NPs, including silver (Ag), aluminium oxide (Al_2_O_3_), CuO, iron oxide (Fe_3_O_4_), gold (Au), magnesium oxide (MgO), nickel (Ni), silicon (Si), SiO_2_, titanium dioxide (TiO_2_), and ZnO, have already been shown to exhibit beneficial antibacterial effects in *in vitro* plant tissue culture (Beyth et al., 2015; Ghasemi et al., 2015; Javed et al., 2017).

In the natural environment, Stevia plants produce bioactive and defense-related secondary metabolites including glycoalkaloids, calystegine alkaloids, steroidal alkaloids, protease inhibitors, lectins, phenolic compounds, flavonoids, and antioxidant enzymes. These bioactive second metabolites have either beneficial effects on the diet or show phytopathogenic effects on plant survival upon infections, as stated by Kovačević et al. (2018).

Silver nanoparticles (AgNPs) have sparked international interest due to their exceptional physiological features, including a better anti-microbial capacity. For example, a simple and green synthesis approach for phytofabrication Zinc oxide-silver supported biochar nanocomposite (Ag/ZnO@BC) using *Persicaria salicifolia* biomass has been studied for the first time to support multiple green chemistry such as less harmful chemical syntheses (Eltaweil et al., 2022; Hosny et al., 2022). Furthermore, AgNPs have been frequently used in plant cell cultures to impact plant cell proliferation, biomass production, and the formation of bioactive secondary metabolites. Nanoparticles are one of the most recently studied elicitors because they have a large impact on physiological processes in plants such as seed germination, growth, biomass, and metabolism. *In vitro*, the addition of AgNPs to the culture medium had a significant influence on *S. rebaudiana* shoot growth (Castro-González et al., 2019).

Many studies on the *in vitro* propagation, multiplication, and callogenesis of *S. rebaudiana* have been undertaken in the past, but there is little information available on callus proliferation and the induction of antioxidants and bioactive chemicals that are helpful to human health (Ahmad et al., 2013; Ahmad et al., 2022).

**FIGURE 6:**
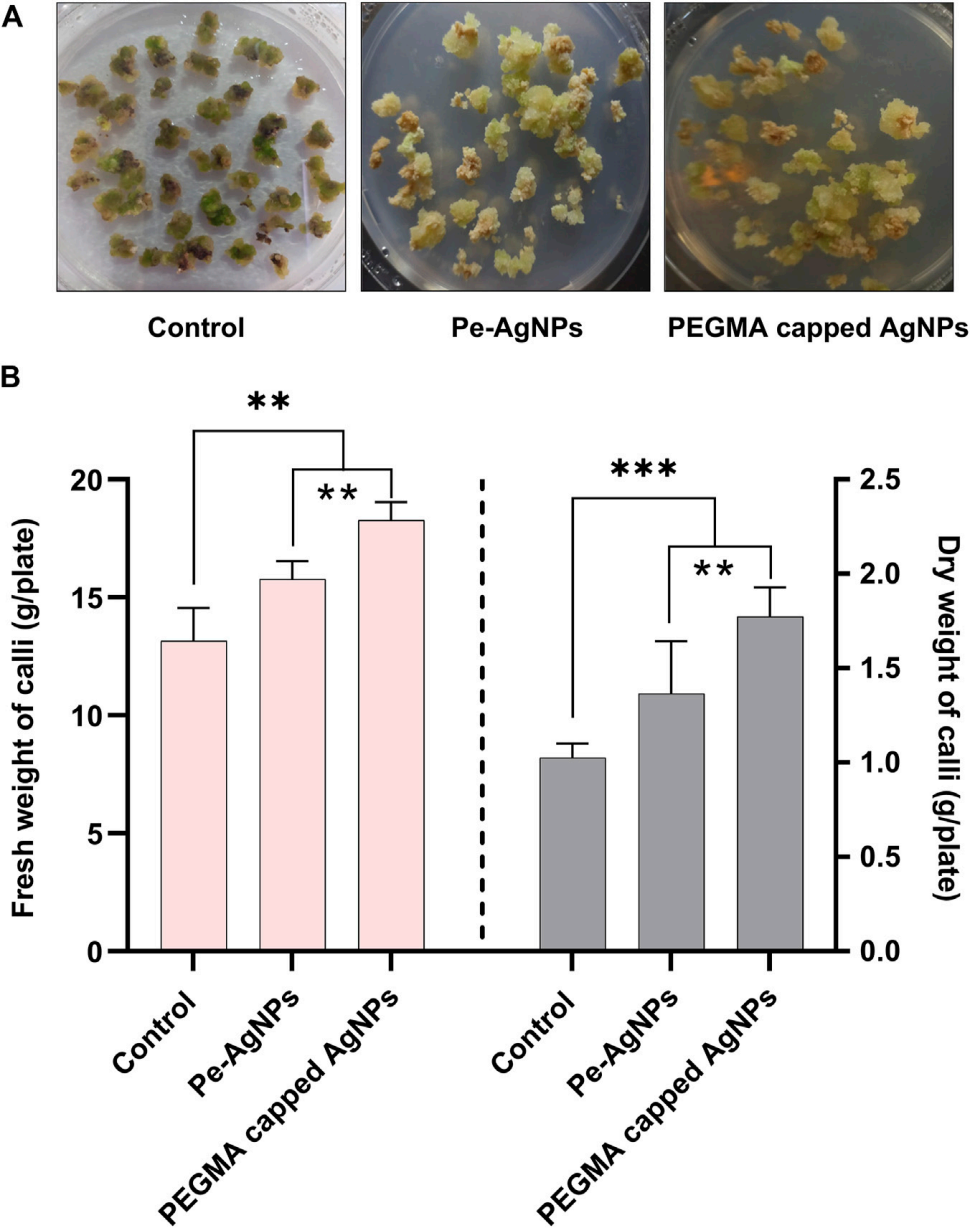
Effects of virgin Pe-AgNPs and PEGMA capped AgNPs *in-vitro* callogenesis response and biomass production of *S. rebaudiana* under a controlled environment, **(A)** Comparison of callus production at 40 DAC, **(B)** Fresh weight (left penal) and dry weight (right penal). Values are the mean ± standard error from three replicates. Asterisks indicate a significant difference (*p* ≤ 0.05) in values between control and treated cultures. FW, Fresh weight, DW, Dry weight, DAC, Days after culturing.

Plant tissue cultures are essential for conservation, bulk propagation, genetic modification, bioactive chemical synthesis, and plant development. Current research revealed *in vitro* callus induction and proliferation responses in terms of callus biomass of *S. rebaudiana* that was significantly enhanced on MS medium fortified with PEGMA capped AgNPs and virgin Pe-AgNPs, compared to control cultures (Figure 6A). The higher fresh and dry weight of steviol glycosides calli was found by supplementing the PEGMA capped AgNPs and virgin Pe-AgNPs compared to control cultures.

Callus biomass in terms of fresh and dry weight was significantly (*p* < 0.05) increased on MS media (2 mg/L NAA+2 mg/L BAP+2 mg/L 2,4-D) fortified with 2 mg L^−1^ virgin Pe-AgNPs (2 mg L^−1^) (0.65 ± 0.004 g) and PEGMA capped AgNPs (0.65 ± 0.004 g) compared to control cultures (MS medium (2 mg/L NAA+2 mg/L BAP+2 mg/L 2,4-D) (0.65 ± 0.004 g). Dry weight was also significantly (*p* < 0.05) increased upon supplementing the virgin Pe-AgNPs (0.65 ± 0.004 g) and PEGMA capped AgNPs (0.082 ± 0.005 g) compared to control (04 ± 0.003 g). However, PEGMA-capped AgNPs proved better than the virgin Pe-AgNPs for the induction of callus.

On day 40, the biomass accumulation was significantly (P 0.05) higher on a medium containing virgin Pe-AgNPs (15.7 g FW/plate; 1.36 g DW/plate), while the maximum increase was found by supplementing PEGMA capped AgNPs (18.21 g FW/plate; 1.77 g DW/plate), compared to the control (13.51 g FW/plate; 1.02 g DW/plate) (Figure 6B).

**FIGURE 7:**
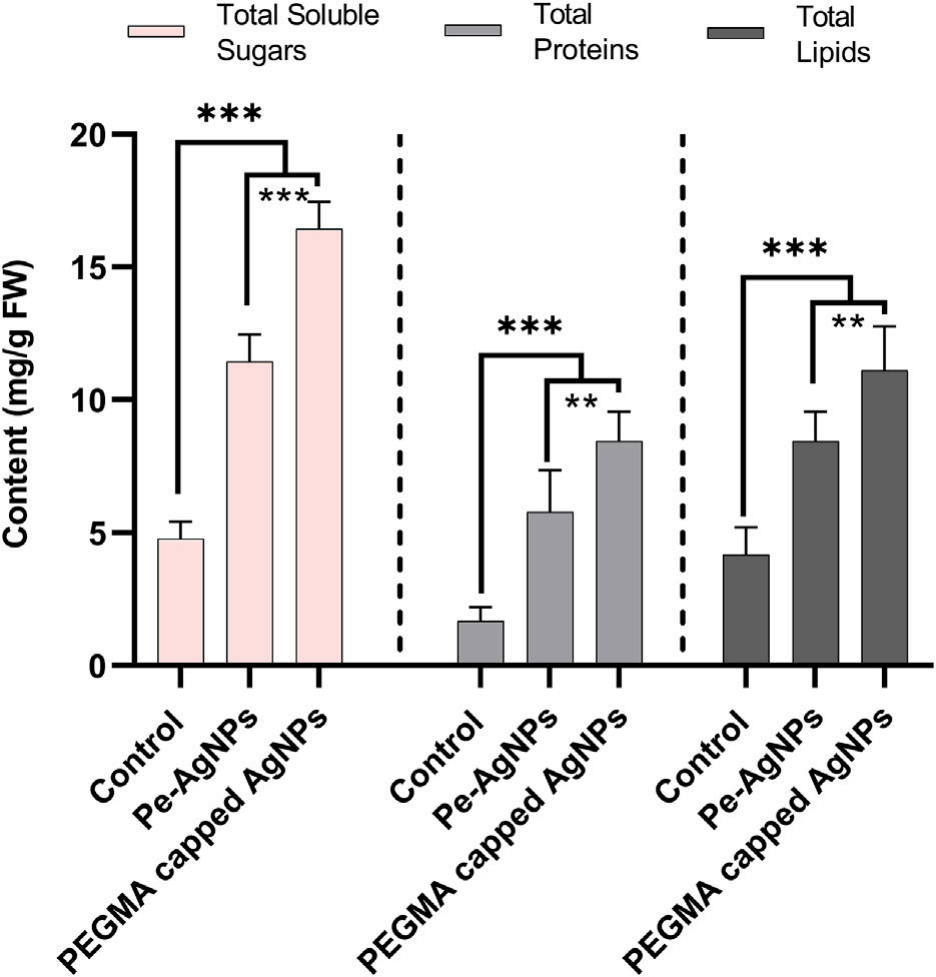
Effects of virgin Pe-AgNPs and PEGMA capped AgNPs on the production of primary metabolites from *in-vitro* grown callus cultures of *S. rebaudiana* under a controlled environment. Total soluble sugars (left penal), total proteins (middle penal), and total lipids (right penal) were measured on the 40th day of incubation. Values are the mean ± standard error from three replicates. Asterisks indicate a significant difference (*p* ≤ 0.05) in values between control and treated cultures. FW, Fresh weight, DAC, Days after culturing.

Forty days after incubation, on a medium containing virgin Pe-AgNPs, significantly (*p* ≤ 0.05) increased total soluble sugars (11.3 mg/g FW) were recorded, while the maximum increase was found by supplementing PEGMA capped AgNPs (16.43 mg/g FW), compared with control (4.7 mg/g FW) (Figure 7). The significantly (*p* ≤ 0.05) increased total proteins (5.7 mg/g FW) was observed on a medium containing virgin Pe-AgNPs on day 40, while the maximum increase was found by supplementing PEGMA capped AgNPs (8.4 mg/g FW), compared with control (1.67 mg/g FW) (Figure 7). On the 40th day after culturing, significantly (*p* ≤ 0.05) increased total lipids (8.4 mg/g FW) were observed on a medium containing virgin Pe-AgNPs, while the maximum increase was found by supplementing PEGMA capped AgNPs (11.1 mg/g FW), compared with control (4.3 mg/g FW) (Figure 7).

**FIGURE 8:**
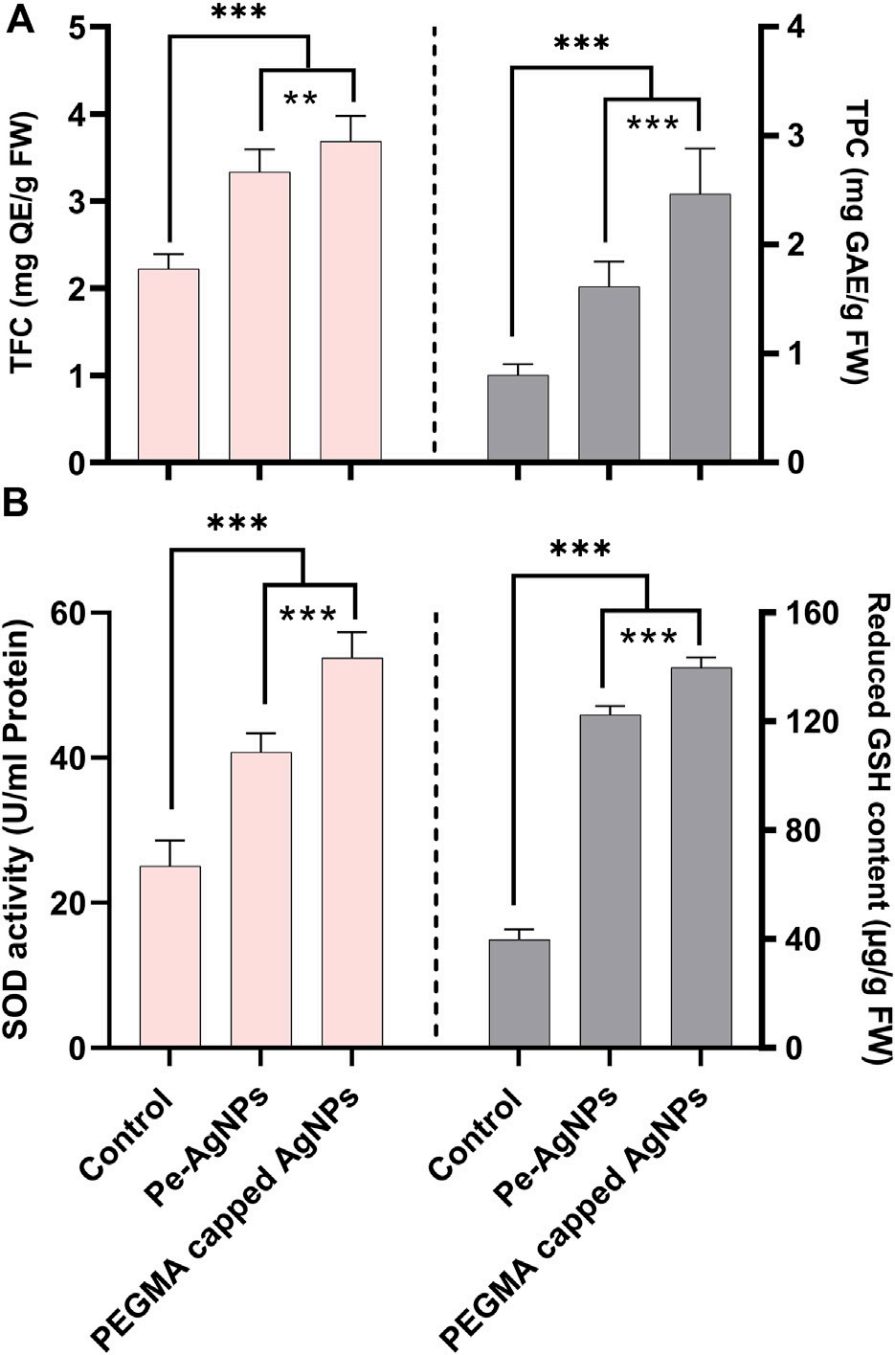
Effects of virgin Pe-AgNPs and PEGMA capped AgNPs on the production of secondary metabolites from *in-vitro* grown callus cultures of *S. rebaudiana* under a controlled environment, **(A)** Total flavonoid content (left penal), total phenolic content (right penal), and **(B)** SOD enzyme activity (left penal) GSH-reduced content (right penal) were measured on the 40th day of incubation. Values are the mean ± standard error from three replicates. Asterisks indicate a significant difference (*p* ≤ 0.05) in values between control and treated cultures. FW: Fresh weight, DAC: Days after culturing.

Forty days after incubation, on a medium containing virgin Pe-AgNPs, significantly (P 0.05) increased total flavonoid content (3.33 mg QE/g-FW) was recorded, while the maximum increase (3.7 mg QE/g-FW) was found by supplementing PEGMA capped AgNPs (3.7 mg QE/g-FW) compared to the control (2.22 mg QE/g-FW) (Figure 8A).

Forty days after incubation, on a medium containing virgin Pe-AgNPs, significantly (P 0.05) higher total phenolic content (1.61 mg GAE/g-FW) was recorded, while the highest increase (2.45 mg GAE/g-FW) found by supplementing PEGMA capped AgNPs (2.45 mg GAE/g-FW) compared to the control (0.8 mg GAE/g-FW) (Figure 8A).

Using AgNPs alone in the growth media or combination with plant growth regulators (PGRs) such as naphthalene acetic acid (NAA) had a significant impact on callus development and antioxidant capability in plant tissue cultures. The activation of the antioxidant response by metallic NPs was employed to promote favorable effects on callus induction, shoot regeneration, and *in vitro* growth (Kim et al., 2017).

In the present research, the callus cultures grown in media supplemented with the virgin Pe-AgNPs significantly (*p* ≤ 0.05) increased the SOD activity (40.75 U/ml protein) on day 40th of incubation, while the maximum increase was found to be more efficient by supplementing PEGMA capped AgNPs (53.78 U/ml protein), compared with control (25.8 U/ml protein) (Figure 8B).

Forty days after incubation, on a medium containing virgin Pe-AgNPs, there was a significantly (P 0.05) increased GSH content (122.5 g/g FW), while the maximum increase was found by supplementing PEGMA capped AgNPs (139.75 g/g FW), compared to the control (39.75 g/g FW) (Figure 8B).

**FIGURE 9:**
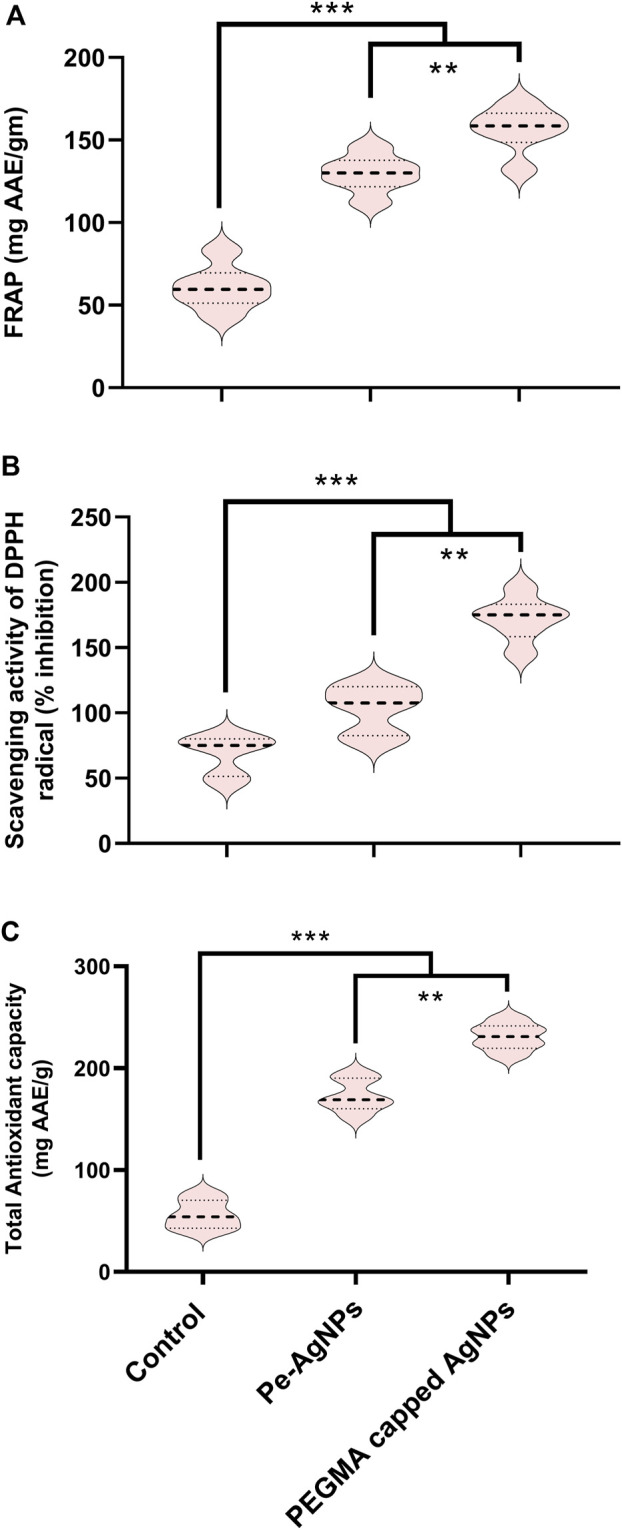
Effects of virgin Pe-AgNPs and PEGMA capped AgNPs on antioxidant potential from *in-vitro* grown callus cultures of *S. rebaudiana* under a controlled environment. **(A)** FRAP, **(B)** DPPH (% free radical scavenging activity), and **(C)** Total antioxidant capacity. Values are the mean ± standard error from three replicates. Asterisks indicate a significant difference (*p* ≤ 0.05) in values between control and treated cultures. FW, Fresh weight, DAC, Days after culturing.

Forty days after incubation, on a medium containing virgin Pe-AgNPs, FRAP (135 mg AAE/g FW) was significantly (P 0.05) increased, while the maximum increase was found by supplementing PEGMA capped AgNPs (156 mg AAE/g FW), compared to the control (54 mg AAE/g FW) (Figure 9A).

Forty days after incubation, on a medium containing virgin Pe-AgNPs, significantly (P 0.05) increased DPPH radical scavenging activity (% inhibition) (110%) was observed, while the maximum increase (176%) found by supplementing PEGMA capped AgNPs (176%) compared to control (79%) (Figure 9B).

Forty days after incubation, on a medium containing virgin Pe-AgNPs, significantly (P 0.05) increased total antioxidants (176 mg AAE/g FW), while supplementing PEGMA capped AgNPs significantly (P 0.05) increased total antioxidants (232 mg AAE/g FW) compared to the control (67 mg AAE/g FW) (Figure 9C).

Our findings indicated that these green synthesized biogenic AgNPs proved to be efficient elicitors for biomass enhancement and beneficial secondary metabolic induction in Stevia callus culture at optimum concentration. However, the PEGMA-capped AgNPs proved better than the virgin Pe-AgNPs for the induction of callus.

## Conclusion

The present study provided a green, one-step, and concise technique for synthesizing AgNPs at atmospheric pressure and ambient temperature. Due to the obvious lower energy use, the entire approach is considered “green” (reaction at atmospheric pressure and room *temperature*)*.* *Periploca aphylla* Dcne. is an efficient source for AgNP fabrication. The UV-vis spectroscopy of a solution containing plant extract and silver nitrate solution revealed the greatest absorbency in the 400–500 nm region, confirming the production of AgNPs. The nanoparticles produced were discovered to be crystalline spherical and biofunctionalized with organic compounds. *Energy* dispersion spectroscopy indicated the confirmation of the presence of the Ag element. The aforesaid study concluded that bio-fabricated, green synthesized AgNPs formulations (virgin Pe-AgNPs and polymer hybrid PEGMA capped AgNPs) considerably demonstrated an emerging use of nanotechnology in agriculture biotechnology for the provision of sustainable biomass production and advantageous secondary metabolite induction *via* rapid *in vitro* callus cultures.

## Data Availability

The raw data supporting the conclusion of this article will be made available by the authors, without undue reservation.
